# The impact of open versus closed format ICU admission practices on the outcome of high risk surgical patients: a cohort analysis

**DOI:** 10.1186/1471-2482-11-18

**Published:** 2011-08-23

**Authors:** Frederik J van der Sluis, Cornelis Slagt, Barbara Liebman, Jan Beute, Jan WR Mulder, Alexander F Engel

**Affiliations:** 1 Department of Surgery of the Zaandam Medical Centre, Zaandam, the Netherlands; 2 Departments of Intensive Care Medicine and Anesthesiology of the Zaandam Medical Centre, Zaandam, the Netherlands

## Abstract

**Background:**

In the year 2000, the organizational structure of the ICU in the Zaandam Medical Centre (ZMC) changed from an open to a closed format ICU. The objective of this study was to evaluate the effect of this organizational change on outcome in high risk surgical patients.

**Methods:**

The medical records of all consecutive high risk surgical patients admitted to the ICU from 1996 to 1998 (open format) and from 2003 to 2005 (closed format), were reviewed. High-risk patients were defined according to the Identification of Risk in Surgical patients (IRIS) score. Parameters studied were: mortality, morbidity, ICU length of stay (LOS) and hospital LOS.

**Results:**

Mortality of ICU patients was 25.7% in the open format group and 15.8% in the closed format group (p = 0.01). Morbidity decreased from 48.6% to 46.1% (p = 0.6). The average length of hospital stay was 17 days in the open format group, and 21 days in the closed format group (p = 0.03).

**Conclusions:**

High risk surgical patients in the ICU are patients that have undergone complex and often extensive surgery. These patients are in need of specialized treatment and careful monitoring for maximum safety and optimal care. Our results suggest that closed format is a more favourable setting than open format to minimize the effects of high risk surgery, and to warrant safe outcome in this patient group.

## Background

Surgical patients form a relatively large portion of the patients admitted to the ICUs in the Netherlands. Approximately 58% of the patients admitted to an ICU in the Netherlands in the year 2007 underwent either urgent or elective surgery before or during ICU admission [1,2].

The outcome of intensive care is determined by multiple factors. Pre-morbid condition and the severity of the presenting health problems have an important effect on outcome.

The majority of postoperative complications and ICU admissions in surgical patients occur in a relative small group of high risk patients [3,4]. Various surgical risk classification systems exist that seek to identify individual patients who may develop perioperative morbidity and mortality [3-5]. For a scoring system to be useful in clinical practice it must be practical, simple to use, discriminative and rely on objective data. The Identification of Risk In Surgical patients (IRIS) score stratifies surgical patients into outcome related groups [4].

It has been shown that structural changes in perioperative management may influence perioperative morbidity and mortality [6-10]. In general two types of ICU staffing can be recognized; "open" and "closed" format [11]. In an open format ICU patients can be admitted to the ICU by any one of a patients physicians. The care for the patient's health status is continued during the ICU stay by this physician. Elective intensivist consultation is possible; however the primary physician has end responsibility concerning treatment decisions. Furthermore, the primary physician has other responsibilities outside the ICU. In a closed format ICU the patients care is transferred to an intensive care physician. This physician is trained in critical care medicine and has no clinical responsibilities outside the ICU. Organizational change to a closed format ICU has been associated with a reduction in mortality and hospital length of stay (LOS) in various patient groups [12].

To our knowledge the effects of ICU format change on the outcome of high risk surgical patients have not been previously described.

The aim of this study was to investigate the effect of an ICU format change from open to closed format, on the outcome of high risk surgical patients.

## Methods

This was a retrospective study. Patient data were collected from an existing hospital database.

Since 1990 data from all general and trauma surgery patients admitted to the Zaandam Medical Centre (ZMC) were prospectively collected in a digital database. The following data were collected: age, sex, type of surgery, specific code of surgical procedure, multiple procedures, acute admission, acute surgery, in hospital morbidity and mortality. Acute surgery was defined as surgery taking place within 24 h after admission. Mortality was defined as in-hospital mortality, i.e. death of any cause during admission. At discharge a dedicated complication form was completed for all patients by the attending ward physician. Morbidity was scored using specific predetermined criteria that have been previously [13] described. Table 1 provides a listing of the complication types and definitions that were scored. High-risk patients were identified using the Identification of Risk In Surgical patients (IRIS) score [4], an in-house developed risk model. The IRIS score predicts postoperative mortality and morbidity in the population of patients operated on in the ZMC. To calculate the IRIS score four parameters are used (table 2).

**Table 1:** Potential complications recorded at hospital discharge

1. Deep wound infection: lay open of the wound was mandatory
2. Intra-abdominal abscess: confirmed by laparotomy or percutaneous drainage
3. Anastomotic leak: confirmed by intraluminal contrast studies and/or laparotomy
4. Re-bleeding or significant wound haematoma
5. Pneumonia: confirmed by chest X-ray and/or mucus cultures
6. Thrombosis and/or pulmonary emboli: confirmed by either venography, duplex sonography and computed tomography scan
7. Pressure ulcer (grade II, III and IV were scored)
8. Urinary tract: infections confirmed by urine cultures
9. Cardiac (acute coronary syndromes, decompensated heart disease, arrhythmias): confirmed by electrocardiography and/or CK/CKMB studies or by chest X-ray
10. Ischemic or hemorrhagic stroke: confirmed by computed tomography scan
11. Sepsis (SIRS caused by an infection)
12. Miscellaneous
13. Multiple organ failure (altered organ function in an acutely ill patient such that homeostasis cannot be maintained without intervention)

**Table 2:** The Identification of Risk in Surgical Patients (IRIS) Score elements

Variable	Points
Age (years)	

0-32	0

33-52	1

53-69	2

> 69	3

Acute admission	

No	0

Yes	1

Acute surgery*	

No	0

Yes	1

Severity of surgery**	

0-3	0

4-7	1

IRIS: Identification of Risk in Surgical patients

* Surgery taking place within 24 h after admission

** Severity of surgery as defined by the Dutch Surgical Society classification of surgical procedures [22]

Patients with an IRIS score of four or higher were considered high risk surgical patients. Two groups were defined: all consecutive high risk surgical patients admitted at the ICU from 1996 to 1998 (open format) and all consecutive high-risk surgical patients admitted at the ICU from 2003 to 2005 (closed format). To eliminate effects associated with the transition these periods were chosen at equal distance from 2000, the year of transition.

Medical records of all eligible patients were reviewed. The reviewer had no prior knowledge on study objective and the different study periods that were chosen. Furthermore, the reviewer was not involved in the medical care that was provided on the ICU. APACHE II scores and APACHE II predicted mortality were calculated. Furthermore the Standardized Mortality Ratios (SMRs) were calculated for both patient groups. The SMR is calculated by dividing the observed mortality to the APACHE II predicted mortality. Values above 1.0 represent an observed mortality that is higher than expected based on the APACHE II predicted mortality.

### Hospital and ICU characteristics

The Zaandam Medical Centre is a teaching hospital serving a population of approximately 145.000 persons in a well defined and stable area. During the study period a 24 h emergency room and operating theatre facilities were available. In this period seven surgeons and ten registrars worked together in the ZMC.

The ICU consisted of a separate department that could provide specialized treatment for the critical ill patient like mechanical ventilation, dialysis and invasive cardiovascular monitoring. The ICU contained seven beds throughout the entire research period. On these beds approximately 2550 patients were admitted per year. During the study period each ICU shift was staffed with four registered ICU nurses during daytime and three during evening/night shifts. In the year 2000, the organizational structure of the ICU in the Zaandam Medical Centre (ZMC) changed from an open format to a closed format ICU. During the open format period critically ill patients in the ICU were cared for by their primary physician. Other medical specialties were involved on a consultant base. No structural multidisciplinary meetings were held. After the shift to a closed format ICU a certified intensivist became responsible for all patients admitted to the ICU. Also during off-hours the ICU became permanently staffed with a physician trained in critical care medicine. End responsibility regarding all treatment decisions in surgical patients shifted from the surgeon to the intensivist. Daily rounds were implemented and ICU nurses were directly supervised by the fulltime intensivist. The ICU became a functional unit (organization and management) within the hospital. Furthermore, structural multidisciplinary meetings consisting of a surgeon, pulmonologist, renal physician, pharmacist and microbiologist were introduced.

After format change, admittance to the ICU was only possible in consultation with an intensivist.

### Statistical analysis

Results were made insightful using the mean value of a parameter for continuous parameters and percentages for dichotomous parameters. Data were analyzed using Statistical Package for the Social Sciences (SPSS) version 14.0 (Boston, MA). Parameters were compared using the Pearson chi-square test and the Student t-test for equality of means. P-values under 0.05 were considered to be significant.

Because the study was conducted in a retrospective fashion and patient data were collected from an existing hospital database, the study received exemption status according to the Zaandam institutional ethical regulations.

## Results

From 1996 to 1998, 230 high risk surgical patients (IRIS ≥ 4) were admitted to the ICU and from 2003 to 2005, 228 patients were admitted. Medical records were retrieved in all patients. The characteristics of both groups are shown in table 3. Patients in the closed format group had a higher mean APACHE II score (mean difference 2.72 points; 95% confidence interval 1.0 to 4.5). Both groups had similar IRIS scores.

**Table 3:** Patient-characteristics

	Open format ICU 1996-1998 (N = 230)	Closed format ICU 2003-2005 (N = 228)	Mean difference (95% confidence interval)	P-value
Gender male	57%	52%		0.23

age	73 (12)	75 (11)	2 (-0.1 - 4.2)	0.06

APACHE II score	14 (6.1)	16 (8.6)	2.7 (1.0-4.5)	0.002

IRIS score	4.73 (0.87)	4.67 (0.87)	0.06 (-0.1 - 0.2)	0.5

Values are mean (sd) unless indicated otherwise. ICU: Intensive Care Unit; APACHE II: Acute Physiology and Chronic Health Evaluation II; sd: standard deviation; IRIS: Identification of Risk in Surgical patients.

The outcome parameters are summarized in table 4. In the closed format group mortality was significantly lower (15.8% in the closed format group versus 25.7% in the open format group, p = 0.01). In the open format group case mix-adjusted mortality was higher compared to the closed format group. The SMR was 1.4 in the open format group compared to 0.7 in the closed format group. The number of patients that developed a complication did not differ significantly between groups. The mean number of complications per patient, was higher in the closed format group (p < 0.01). Cardiopulmonary complications were observed in 21.8% of patients in the open format group and 25.9% of patients in the closed format group (p = 0.32). Mortality due to a cardiopulmonary complication was higher in the open format group (12.2% in the open format group versus 8.3% in the closed format group, p = 0.02). Total LOS and ICU LOS were significantly longer in the closed format group (table 5). Furthermore, survivors spent significantly more days on the ICU after format change.

**Table 4:** Patient outcome results

	Open format ICU 1996-1998 (N = 230)	Closed format ICU 2003-2005 (N = 228)	P-value
Mortality	25.7%	15.8%	0.01

APACHE predicted mortality	18.6%	23.5%	0.03

Morbidity	48.7%	46.1%	0.6

Complications per patient (mean, sd)	1.67 (0.94)	2.09 (1.28)	< 0.01

Cardiopulmonary complications	21.8%	25.9%	0.32

Mortality due to cardio- pulmonary complications	12.2%	8.3%	0.02

Values are percentages unless indicated otherwise. ICU: Intensive Care Unit; sd: standard deviation.

**Table 5:** Length of stay results

	Open format ICU 1996-1998 (N = 230)	Closed format ICU 2003-2005 (N = 228)	Mean difference (95% confidence interval)	P-value
Hospital LOS:				

Overall	16.9 (17.5)	21.2 (24.3)	4.2 (0.2-8.3)	0.04

Survivors	18.3 (15.0)	21.9 (25.0)	3.6 (-0.9-8.0)	0.12

Non-survivors	12.4 (23.6)	16.8 (19.4)	4.4 (-5.9-14.7)	0.40

ICU LOS:				

Overall	2.5 (4.1)	4.8 (9.3)	2.2 (0.9-3.6)	< 0.01

Survivors	1.7 (2.7)	4.3 (8.5)	2.6 (1.2-3.9)	< 0.01

Non-survivors	5.0 (6.2)	7.4 (12.4)	2.4 (-1.4-6.2)	0.21

Values are mean (sd) unless indicated otherwise. ICU: Intensive Care Unit; sd: standard deviation; LOS: length of stay.

## Discussion

This study has shown that changing the organizational structure of an ICU from open to closed format is associated with a reduction of postoperative mortality in high risk surgical patients.

Before format change patients could be admitted to the ICU by any one of a patient's physicians. During the patients ICU stay the care for the patients' health status was continued by this physician. After format change, admittance to the ICU was only possible in consultation with an intensivist. Patient care transferred from the primary physician to the intensivist. This intensivist had no clinical responsibilities outside the ICU. Furthermore, daily rounds by the attending intensivist were introduced and the ICU became permanently staffed with an intensivist during off-hours.

High risk surgical patients admitted to the ICU after format change had a higher mean APACHE II score. This implies that patients in the closed format group had a more severe disease and a higher risk of death. Another possible explanation for the difference in mean APACHE II score is the difference in mean age between the groups. Despite of the difference in mean APACHE II score, mortality decreased. This is reflected by the decrease in SMR after format change. This strengthens the observation that closed format ICU treatment is associated with reduced postoperative mortality in high risk surgical patients. The differences in baseline disease severity may be explained by a difference in admittance threshold between intensivists and physicians without critical care training. It appears that physicians without training in critical care medicine tend to apply a lower admittance threshold. Because of this, changing the format of an ICU may lead to a reduction of inappropriate ICU admissions and its associated costs. Another possible explanation for the increase in mean APACHE II score is the gradual change in demographic characteristics. The proportion of adults and elderly in the population is gradually increasing [14]. This phenomenon causes the number of elderly patients with serious co-morbid conditions in need for surgery to increase. Since age is associated with a rise in pre-morbid conditions, the proportion of high risk surgical patients increases.

Although the severity of disease was higher in the closed format group, we found that mortality decreased significantly after format change. This finding is in accordance with the results of similar observational studies performed in various populations [15-18].

The decline in mortality was also observed in patients that developed cardiopulmonary complications. It appears that cardiopulmonary complications were better treated after format change. This may be explained by the immediate on-site availability of an intensivist. The introduction of having daily rounds by a physician trained in critical care medicine might allow for earlier diagnosis and prompt treatment of the specific set of complications seen in the critically ill patient. However the current study does not provide any evidence for this statement.

Most studies on ICU format change and physician staffing patterns observe a decrease in total and ICU LOS [12]. In contrast to these studies we observed an increase in total and ICU LOS. This may partly be explained by the difference in baseline severity of disease between the open and closed format group. Also improved treatment of severe complications may have resulted in an improved survival at the cost of a longer ICU stay.

With advances in intensive care medicine, caring for the critically ill has become more complicated. This has led to the discussion; who should care for the ICU patient. Many, mainly observational studies, have been executed to evaluate the quality of care in ICUs. Staffing ICUs with Intensive Care physicians and directing all care to the Intensive Care physician is associated with reduced hospital and ICU mortality [12]. Other ICU characteristics that may also be associated with improved patient outcome are; increased nurse patient ratios [15], having daily rounds by an intensivist [12,15,19] and the use of computerized warning and monitoring systems [20,21].

The retrospective analysis is a shortcoming of the present study. However, since a prospective database was used, specifically designed to document complications and criteria to score complications were protocolized and did not change over time, the quality of outcome data seems robust.

The present study compares outcome data that were obtained in different time periods. Medicine has developed, in particular, intensive care medicine and surgical procedures/care/technology. Extensive surgery has become possible at increasing age. Advanced insight of the perioperative care like fluid management, temperature, stress reduction, perioperative beta blockers, postoperative pain reduction have progressed over the study period. Furthermore, new antimicrobials/antifungals, improved glycemic control and transfusion practices became available during the study period. Advances in surgical technique, perioperative care, general and intensive care medicine may reduce postoperative mortality over time. This temporal trend in mortality reduction has biased the study results. We attempted to address the magnitude of this bias by looking at nationwide trends of mortality reduction for the general Dutch ICU population. The Dutch National Intensive Care Evaluation was founded in 1996. Over the years that followed an increasing number of ICUs started exporting data concerning severity of disease and outcome to a national database. When looking at nationwide trends a gradual decrease in SMR is observed for the general Dutch ICU population. In the year 1999 the SMR was 1.03 on a national level which decreased to 0.87 in the year 2004 [2]. Although the study demonstrated a much larger decrease in SMR (1.4 to 0.7), it was not designed to distinguish between the general temporal trend and mortality reduction due to format change. However, since format change was a major change in this period and a proportionally large reduction in mortality was observed, format change is likely to have played a major role in mortality reduction.

## Conclusions

The present data suggests that closed format ICU organization is associated with improved outcome in high-risk surgical patients. Especially patients with cardiopulmonary complications appear to benefit from closed format ICU organization.

The observed decrease in mortality can partly be explained by advances in medicine, however when looking at nation wide trends, organizational change from open to closed format ICU is likely to have played an important role.

## List of Abbreviations

**APACHE II: **Acute Physiology and Chronic Health Evaluation II; **ICU: **Intensive Care Unit; **IRIS score: **Identification of Risk in Surgical patients score; **LOS: **length of stay; **Sd: **standard deviation; **SMR: **Standardized Mortality Ratio; **SPSS: **Statistical Package for the Social Sciences; **ZMC: **Zaandam Medical Centre.

## Competing interests

The authors declare that they have no competing interests.

## Authors' contributions

AE and JB conceived of the study and its design. BL was responsible for acquisition and formatting of the data. CS was involved in drafting and revising the manuscript. FS analyzed the data and wrote the manuscript. JM revised the manuscript. AE coordinated the project and helped to interpret the data. All authors read and approved the final manuscript.

## Pre-publication history

The pre-publication history for this paper can be accessed here:

http://www.biomedcentral.com/1471-2482/11/18/prepub
